# Long-term molecular surveillance of *Cryptosporidium* and *Giardia* in wildlife in protected drinking water catchments

**DOI:** 10.1186/s13071-025-07048-8

**Published:** 2025-10-14

**Authors:** Anson V. Koehler, Tao Wang, Melita A. Stevens, Shane R. Haydon, Robin B. Gasser

**Affiliations:** 1 Department of Veterinary Biosciences, Melbourne Veterinary School, Faculty of Science, The University of Melbourne, Parkville, VIC 3010 Australia; 2 Melbourne Water Corporation, Docklands, VIC 3008 Australia

**Keywords:** *Cryptosporidium*, *Giardia*, Wildlife, Protected drinking water catchments, Long-term monitoring, Molecular tools

## Abstract

**Background:**

This study presents findings from a 15-year longitudinal surveillance program (2009–2024) monitoring *Cryptosporidium* and *Giardia* in protected drinking water catchments in Melbourne and environs in the State of Victoria, Australia. As one of the few major cities worldwide sourcing largely unfiltered water from forested catchments, Melbourne presents a unique opportunity to assess the occurrence and prevalence of protozoan parasites in a minimally disturbed ecosystem.

**Methods:**

A total of 14,960 animal faecal samples were analysed using polymerase chain reaction (PCR)-based sequencing, including 8695 samples collected over the past 9 years.

**Results:**

*Cryptosporidium* was detected in 3.15% of samples and *Giardia* in 0.16%. A total of 12 recognised *Cryptosporidium* species and genotypes were identified, nine of which have known zoonotic potential, as well as two sub-assemblages (AI and AIII) of *Giardia duodenalis*, including four novel assemblage AI variants. Parasite diversity was the highest in eastern grey kangaroos, which hosted at least 18 *Cryptosporidium* variants. Temporal analyses revealed significant inter-annual variation, with peak prevalence during the 2023 La Niña year and seasonal differences by host group. Notably, *C. ubiquitum*, *C. muris* and *C. occultus* were recorded for the first time in these catchments. In spite of the low prevalence of high-risk species such as *C. parvum* and the absence of *C. hominis*, the detection of emerging and previously uncharacterised genotypes emphasises the importance of sustained surveillance.

**Conclusions:**

These findings have broad implications for managing zoonotic risk in unfiltered water systems worldwide. Advances in metagenomics and high-throughput sequencing platforms will be critical for enhancing future pathogen monitoring and catchment management strategies in the context of increasing climate and environmental pressures.

**Graphical Abstract:**

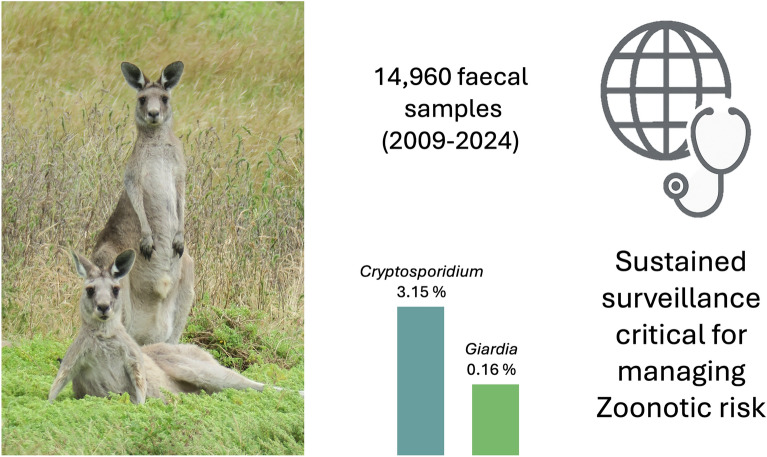

**Supplementary Information:**

The online version contains supplementary material available at 10.1186/s13071-025-07048-8.

## Background

The supply of fresh drinking water to people and the prevention of waterborne diseases are a major challenge, particularly in disadvantaged communities [1]. Worldwide, diarrhoeal diseases are the third leading cause of death and malnutrition in children under 5 years of age, equating to ~1.7 billion cases per annum [2–4]. Globally, *Cryptosporidium* alone accounts for a disease burden of 12.9 million disability-adjusted life-years (DALYs) [5], ranking second only to rotavirus as cause of severe diarrhoea in young children [6]. Together, *Cryptosporidium* and *Giardia* are responsible for most protozoal waterborne disease outbreaks worldwide [7].

Currently, there are 46 recognised species of *Cryptosporidium* and more than 120 genotypes [8, 9]. Of these, 19 species and four genotypes have been reported in humans, with *C. parvum* and *C. hominis* accounting for 95% of all *Cryptosporidium* species described to date [8]. There are more than eight phylogenetically distinct species of *Giardia* [10]. *Cryptosporidium* and *Giardia* are unique in that very small numbers of infective stages (oocysts and cysts, respectively) can lead to disease in humans [11, 12], and these stages are resistant to chlorination and other common water treatments [13]. Although outbreaks of cryptosporidiosis can have major adverse human health and economic impacts [14, 15], sub-clinically infected (asymptomatic) people and animals represent key reservoirs for transmission to susceptible or immune-suppressed or deficient people. This context emphasises the major public health importance of waterborne cryptosporidiosis and giardiasis and the need for sustained and effective prevention of these diseases.

Melbourne in the State of Victoria, Australia, is one of the few cities in high-income countries receiving largely unfiltered, chlorinated drinking water from protected wilderness catchment areas [16]. All water from these protected catchments is disinfected with chlorine before it is provided to the city. Each day, approximately 1250 million litres of drinking water are supplied to the more than 5 million residents of Greater Melbourne [16]. The management of these water catchment areas includes restricted access for humans, long water retention times and an intense program of testing and monitoring for pathogens in source water [16]. These catchments represent habitat for native and feral animals, such that the monitoring of zoonotic pathogens is central to management and the rigorous protection of the safety of drinking water [16].

Within the context of an integrated catchment management (ICM) framework, we have been monitoring *Cryptosporidium* and *Giardia* in faecal samples from various mammals and birds in Melbourne’s catchments [17, 18]. Since 2009, we consolidated a joint longitudinal program to consistently monitor (every 1–2 months) the presence of *Cryptosporidium* genotypes and subtypes as well as *Giardia* assemblages in faecal samples from animals in key catchments using molecular tools. All findings are reported to Melbourne Water, which reports to the Department of Health (DH) of the State of Victoria, Australia. Some of the findings produced have been published previously [17–21]. Here, we report the results from December 2015 to July 2024 and also review trends seen over the 15-year period since the beginning of the sample collection program in June of 2009.

## Methods

### Melbourne’s water catchments

**Fig. 1:**
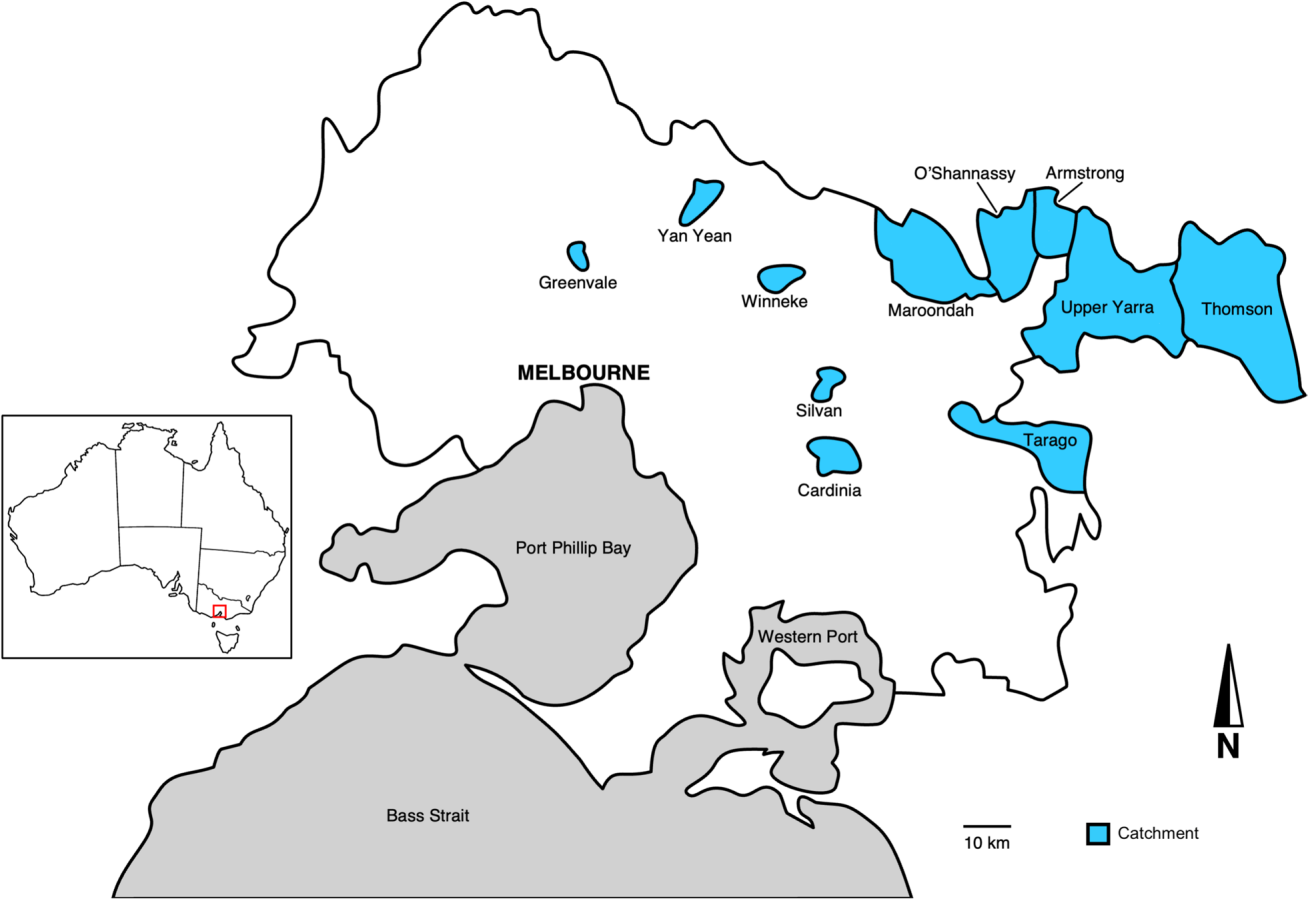
Map of Melbourne Water’s protected drinking water catchments (in blue)

Melbourne’s closed drinking water catchments, designed to minimise the risk of waterborne contamination by restricting domestic animal and human access, are primarily located in the Yarra Ranges, 40–90 km east of Melbourne, spanning more than 150,000 hectares of eucalypt forests. Additional off-stream reservoirs are located closer to the city of Melbourne (Fig. 1). All water is treated in accordance with national and international guidelines [22]. A total of ten major reservoirs and numerous weirs serve the city. The faecal sampling reported here has occurred from most of the catchments for these reservoirs and weirs. The Armstrong weir (AR), Maroondah reservoir (MR), O’Shannassy reservoir (OS), Thomson reservoir (TH) and Upper Yarra reservoir (UY) are situated in the dense eucalypt forests of the Yarra Ranges catchment. The remaining reservoirs – Yan Yean (YY), Cardinia (CA), Greenvale (GV) and Silvan (SV) – primarily function as off-stream storages for the larger catchments and are surrounded by eucalypt and/or pine forests. It should be noted that Winneke (WI) is a large conventional water treatment plant supplied from the off-stream Sugarloaf reservoir, which is in turn filled by pumping from a large multi-use catchment. Samples for this site are from around the treatment plant, not the Sugarloaf catchment. In total, 14,960 samples were collected and tested (from June 2009 to July 2024). Data sets from previous studies [17, 18] were used for selective, comparative analyses.

### Sampling and isolation of genomic DNA

In the context of our *Cryptosporidium* and *Giardia* monitoring program, faecal samples have been – and continue to be – collected from forested or grassy areas in close proximity to water reservoirs – where wildlife typically graze. From December 2015 to July 2024, 8695 faecal deposits from *Wallabia bicolor* (swamp wallaby), *Macropus giganteus* (eastern grey kangaroo), *Vombatus ursinus* (common wombat), *Phascolarctos cinereus* (koala), *Trichosurus vulpecula* (common brushtail possum), *Rusa unicolor* (sambar deer), *Cervus elaphus* (red deer), *Dama dama* (fallow deer), *Oryctolagus cuniculus* (rabbit), *Mastacomys fuscus* (broad-toothed rat), *Rattus fuscipes* (bush rat), *Rattus lutreolus* (swamp rat), *Canis familiaris* (dog), *Canis familiaris dingo* (dingo), *Vulpes vulpes* (red fox), *Dromaius novaehollandiae* (emu) and *Chenonetta jubata* (wood duck) and samples of unknown host origin were collected from nine locations. Specifically, samples were collected from AR (*n* = 15), CA (*n* = 1902), GV (*n* = 1188), MR (*n* = 1482), OS (*n* = 302), SV (*n* = 1489), UY (*n* = 620), WI (*n* = 366) and YY (*n* = 1331) (Fig. 1). It is important to note that some animal species are not evenly distributed across catchments. For instance, fallow deer and emus are seen only in Cardinia, while Greenvale is home to kangaroos, rabbits and foxes. Scats were initially identified using a field guide [23].

From aliquots (~50 mg) of individual faecal samples, genomic DNA was extracted using the PowerSoil kit (MoBio Powersoil Kit; 2015 to April 2021), later rebranded as the DNeasy PowerSoil Pro kit (Qiagen; May 2021 to July 2024) following the manufacturers’ instructions. The specific identity of animals from which individual samples originated was verified by polymerase chain reaction (PCR)-based sequencing of a region of the mitochondrial cytochrome b (*cytb*) gene from faecal DNA using an established method [24]. For some analyses, data for samples from three species of deer (i.e. sambar, red and fallow) were pooled (designated as ‘deer’), as for samples from eastern grey kangaroos and swamp wallabies (designated ‘macropods’).

### PCR-coupled sequencing, sequence alignments and associated analyses

During the monitoring program (from 2015 to 2024), distinct genetic loci have been used to identify *Cryptosporidium* and *Giardia* species via nested PCR-based sequencing (reagents and cycling conditions are presented in Table 1). For the identification of *Cryptosporidium* species, small subunit ribosomal ribonucleic acid (RNA) gene (*SSU*) sequences (800–830 bp) were obtained using well-established methods [18, 21, 25–28]. On occasions, large subunit ribosomal RNA gene (*LSU*) sequences (500 bp) were obtained using another technique [29]. For the differentiation of *C. parvum*, *C. hominis* and *C. cuniculus*, a 60-kDa glycoprotein (*gp*60) gene region (1000 bp) was sequenced using an established approach [18, 30, 31]. For the identification and differentiation of *Giardia* species and assemblages, a triose-phosphate isomerase (*tpi*) gene region (530 bp region) was amplified and sequenced using a published methodology [17, 18, 32]. Known test-positive, test-negative and no-template (including ‘carry-over’) controls were included in each step of each set of PCRs. Bidirectional sequencing of amplicons was conducted by Macrogen (South Korea) or the Australian Genome Research Facility (AGRF; Melbourne).

**Table 1:** The primers and conditions used to obtain *Cryptosporidium* and *Giardia*

Region	PCR	Primers	Size (bp)	Denaturation/annealing/extension	References
*LSU*	1°	LSU2040F—5′ CGAATAGCGTTATCTTTGCTATTT 3′	500	94 °C/30 s 58 °C/30 s 72 °C/50 s 35 cycles	[29]
		LSU3020R—5′ GTCTTCCGCGAAGATCAG 3′			
	2°	LSU2065F—5′ TTACCATGGAAT(C/T)AGTTCAGC 3′		94 °C/30 s 58 °C/30 s 72 °C/50 s 35 cycles	
		LSU2557R—5′ AACACCATTTTCTGGCCATC 3′			
*SSU*	1°	18SiCF2—5′ GACATATCATTCAAGTTTCTGACC 3′	800	94 °C/30 s 58 °C/30 s 72 °C/30 s 45 cycles	[26]
		18SiCR2—5′ CTGAAGGAGTAAGGAACAACC 3′			
	2°	18SiCF1—5′ CCTATCAGCTTTAGACGGTAGG 3′		94 °C/30 s 58 °C/30 s 72 °C/30 s 45 cycles	
		18SiCR1—5′ TCTAAGAATTTCACCTCTGACTG 3′			
*SSU*	1°	SSU-F2—5′ TTCTAGAGCTAATACATGCG 3′	830	94 °C/45 s 55 °C/45 s 72 °C/60 s 35 cycles	[25, 71, 86]
		SSU-R2—5′ CCCATTTCCTTCGAAACAGGA 3′			
	2°	SSU-F3—5′ GGAAGGGTTGTATTTATTAGATAAAG 3′		94 °C/45 s 55 °C/45 s 72 °C/60 s 35 cycles	
		SSU-R4—5′ CTCATAAGGTGCTGAAGGAGTA 3′			
*gp*60	1°	gp15-ATG—5′ ATGAGATTGTCGCCTCATTATC 3′	1000	94 °C/30 s 55 °C/45 s 72 °C/60 s 35 cycles	[31]
		gp15-STOP—5′ TTACAACACGAATAAGGCTGC 3′			
	2°	gp15-15A—5′ GCCGTTCCACTCAGAGGAAC 3′		94 °C/30 s 55 °C/30 s 72 °C/30 s 35 cycles	
		gp15-15E—5′ CCACATTACAAATGAAGTGCCGC 3′			
*tpi*	1°	AL3543—5′ AAATTATGCCTGCTCGTCG 3′	530	94 °C/45 s 50 °C/45 s 72 °C/60 s 35 cycles	[32]
		AL3546—5′ CAAACCTTTTCCGCAAACC 3′			
	2°	AL3544—5′ CCCTTCATCGGTGGTAACTT 3′		94 °C/45 s 50 °C/30 s 72 °C/60 s 35 cycles	
		AL3545—5′ GTGGCCACCACTCCCGTGCC 3′			

DNA sequence data obtained were matched to sequences in the GenBank database using the Basic Local Alignment Search Tool (BLAST; www.ncbi.nlm.nih.gov) and subsequently aligned to curated sequences representing all known distinct *Cryptosporidium* species/genotypes and *Giardia* species/assemblage as well as outgroup taxa in our in-house sequence repository. Pairwise sequence comparisons were conducted, and sequence identities were calculated using Geneious Prime, version 2024.0.7, software (www.geneious.com). Subsequently, sequences were aligned using the MAFFT program [33] and alignments manually adjusted in Mesquite, version 3.81 [34].

Phylogenetic analysis of sequence data was performed by Bayesian inference (BI) using Monte Carlo Markov Chain (MCMC) analysis in MrBayes, version 3.2.7 [35]. Likelihood parameters for the BI analysis of *SSU* data were selected on the basis of the Akaike information criterion (AIC) test in MEGA11 [36], with the general time-reversible (GTR) model having the lowest AIC score. The number of substitutions model (Nst) was set at six, with a gamma distribution and a proportion of invariable sites. Posterior probability (pp) values were calculated after 10,000,000 generations with four simultaneous tree-building chains, saving trees at every 100th generation. At the end of each run, convergence was confirmed by a standard deviation of split frequencies < 0.01, and the potential scale reduction factor approached 1. A 50% majority-rule consensus tree was constructed from the final 75% of trees generated. Each analysis was run three times to ensure convergence and insensitivity to priors. The outgroup included in the analysis was *C. abrahamseni*. Additional statistical analyses were performed using R Statistical Software (version 4.5.1), with graphs generated using ggplot2, dplyr and tidyr packages.

## Results

### Identification and classification of *Cryptosporidium* on the basis of *SSU*

**Fig. 2:**
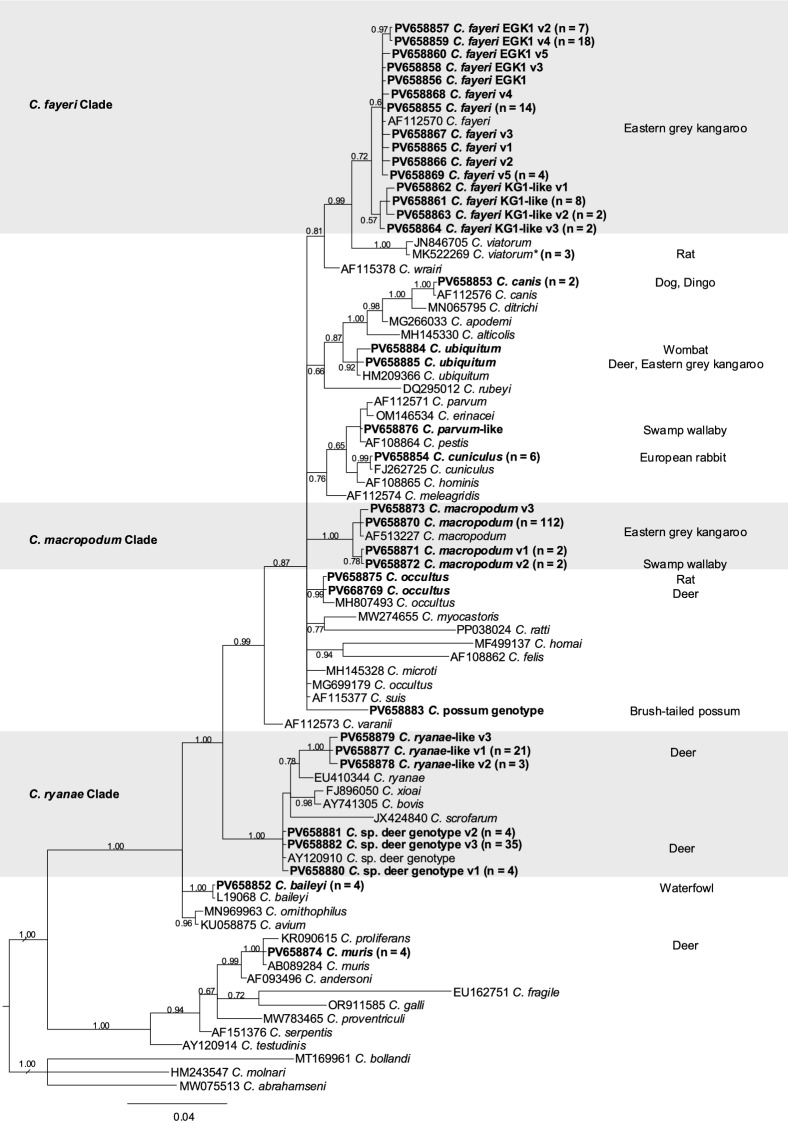
Phylogenetic relationships among *Cryptosporidium* taxa inferred from Bayesian inference analysis of partial small subunit ribosomal RNA (*SSU*) gene sequences. Posterior probabilities are shown at all major nodes. Taxa in bold represent *Cryptosporidium* species or genotypes identified from faecal DNA samples in this study. The number of samples, genotype and corresponding GenBank accession numbers are provided in parentheses. Shaded regions represent the three major clades – *C. fayeri*, *C. ryanae* and *C. macropodum*. The scale bar indicates the number of substitutions per site. *Cryptosporidium abrahamseni* was used as an outgroup

From 8695 faecal DNA samples (prevalence: 3.15%), 274 *SSU* amplicons were obtained, and 13 species and genotypes of *Cryptosporidium* were identified (Additional File 1: Supplementary Table S1). In total, 35 unique sequences were assigned GenBank accession numbers (PV658852 to PV658885 and PV668769) (Table 2) and used for phylogenetic analysis (Fig. 2). In total, we obtained 23 novel *SSU* sequences (i.e. with < 100% identity to sequences in GenBank) – not previously published or deposited in public databases. Each sequence was linked to a host species and water catchment area, and its prevalence was recorded (Additional File 1: Supplementary Table S1). The final designation of species and genotype was based on the alignment and position of a sequence within the phylogenetic tree (Fig. 2). In this tree, most sequences were assigned to the three predominant clades – i.e. *C. ryanae*, *C. fayeri* and *C. macropodum*; the remaining sequences were external to these clades (Fig. 2). In the following sections, we explore *Cryptosporidium* species and genotypes according to host group.

**Table 2:** Summary of *Cryptosporidium* taxa (species and genotypes) identified in faecal samples collected from animals in Melbourne’s protected drinking water catchment areas (December 2015 to July 2024)

Species/genotype	Total	Designation	Subtotal		Human reports^*^	GenBank no.
*Cryptosporidium baileyi*	4	*Cryptosporidium baileyi*	4		None	PV658852
*Cryptosporidium canis*	2	*Cryptosporidium canis*	2		Many	PV658853
*Cryptosporidium cuniculus*	6	*Cryptosporidium cuniculus*	6		Many	PV658854
*Cryptosporidium fayeri*	14	*Cryptosporidium fayeri*	63		Few	PV658855
*Cryptosporidium fayeri* EGK1	1	*Cryptosporidium fayeri*				PV658856
*Cryptosporidium fayeri* EGK1 v2	7	*Cryptosporidium fayeri*				PV658857
*Cryptosporidium fayeri* EGK1 v3	1	*Cryptosporidium fayeri*				PV658858
*Cryptosporidium fayeri* EGK1 v4	18	*Cryptosporidium fayeri*				PV658859
*Cryptosporidium fayeri* EGK1 v5	1	*Cryptosporidium fayeri*				PV658860
*Cryptosporidium fayeri* KG1-like	8	*Cryptosporidium fayeri*				PV658861
*Cryptosporidium fayeri* KG1-like v1	1	*Cryptosporidium fayeri*				PV658862
*Cryptosporidium fayeri* KG1-like v2	2	*Cryptosporidium fayeri*				PV658863
*Cryptosporidium fayeri* KG1-like v3	2	*Cryptosporidium fayeri*				PV658864
*Cryptosporidium fayeri* v1	1	*Cryptosporidium fayeri*				PV658865
*Cryptosporidium fayeri* v2	1	*Cryptosporidium fayeri*				PV658866
*Cryptosporidium fayeri* v3	1	*Cryptosporidium fayeri*				PV658867
*Cryptosporidium fayeri* v4	1	*Cryptosporidium fayeri*				PV658868
*Cryptosporidium fayeri* v5	4	*Cryptosporidium fayeri*				PV658869
*Cryptosporidium macropodum*	112	*Cryptosporidium macropodum*	117		None	PV658870
*Cryptosporidium macropodum* v1	2	*Cryptosporidium macropodum*				PV658871
*Cryptosporidium macropodum* v2	2	*Cryptosporidium macropodum*				PV658872
*Cryptosporidium macropodum* v3	1	*Cryptosporidium macropodum*				PV658873
*Cryptosporidium muris*	4	*Cryptosporidium muris*	4		Many	PV658874
*Cryptosporidium occultus*	1	*Cryptosporidium occultus*	2		Several	PV658875
*Cryptosporidium occultus*	1	*Cryptosporidium occultus*				PV668769
*Cryptosporidium parvum*-like	1	*Cryptosporidium parvum*-like	1		Many	PV658876
*Cryptosporidium ryanae*-like v1	21	*Cryptosporidium ryanae*	68		None	PV658877
*Cryptosporidium ryanae*-like v2	3	*Cryptosporidium ryanae*				PV658878
*Cryptosporidium ryanae*-like v3	1	*Cryptosporidium ryanae*				PV658879
*Cryptosporidium* sp. deer genotype v1	4	*Cryptosporidium ryanae*				PV658880
*Cryptosporidium* sp. deer genotype v2	4	*Cryptosporidium ryanae*				PV658881
*Cryptosporidium* sp. deer genotype v3	35	*Cryptosporidium ryanae*				PV658882
*Cryptosporidium* sp. possum genotype	1	*Cryptosporidium* sp. possum genotype	1			PV658883
*Cryptosporidium ubiquitum* cf C3604	1	*Cryptosporidium ubiquitum*	3			PV658884
*Cryptosporidium ubiquitum* cf W13491	2	*Cryptosporidium ubiquitum*				PV658885
*Cryptosporidium viatorum*	3	*Cryptosporidium viatorum*	3		Many	MG021320
Total	274			^*^According to ref. [8]

### *Cryptosporidium* in deer

Within the *C. ryanae* clade (Fig. 2), four *SSU* sequence variants representing *C. ryanae* and three representing ‘*Cryptosporidium* sp. deer genotype’ [37] were recorded for 66 of a total of 1774 samples from deer (Fig. 2; Tables 2 and 3). *Cryptosporidium* sp. deer genotype v3 was the most prevalent, and recorded in 35 samples, followed by *C. ryanae*-like v1 in 16 samples; three were classified as *C. ryanae*-like using *LSU* [29] but were not characterised by *SSU* and, therefore, were not included in the phylogeny (Fig. 2). The majority of these 66 records were from sambar deer (*n* = 49), followed by red deer (*n* = 7) and fallow deer (*n* = 3) (Additional File 1: Supplementary Table S1); seven were from deer of which species identity was not determined by molecular means [24]. In addition, *C. muris* was detected once in sambar deer in CA and YY catchments in 2021 and again at YY in 2023; *C. ubiquitum* (accession no. PV658885) and *C. occultus* (accession no. PV668769) were each detected once in sambar deer in the UY catchment.

**Table 3:** Summary of *Cryptosporidium* taxa detected in 8695 faecal samples from animals in Melbourne’s protected drinking water catchments (2015–2024)

Catchment	Macropod	Deer	Rabbit	Canid	Rat	Waterbird	Possum	Wombat	*Cryptosporidium* total	Faecal total
Armstrong									0	15
Cardinia	19 *macropodum*, 6* fayeri*	13 *ryanae*, 1 *muris*		1 *canis*					40	1902
Greenvale	36 *macropodum*, 12 *fayeri*			1 *canis*					49	1188
Maroondah	11 *macropodum*, 13 *fayeri*	4 *ryanae*							28	1482
O’Shannassy	1 *parvum*-like	12 *ryanae*	1* cuniculus*						14	302
Silvan	5 *fayeri*, 2* macropodum*		4* cuniculus*			4 *baileyi*			15	1489
Upper Yarra		17 *ryanae*, 1 *occultus* 1 *ubiquitum*			3 *viatorum*, 1 *occultus*			1* ubiquitum*	24	620
Winneke	22 *macropodum*, 1 *ubiquitum*								23	366
Yan Yean	27 *fayeri*, 27* macropodum*	22 *ryanae*, 3 *muris*	1* cuniculus*				1 possum genotype		81	1331
Total	182	74	6	2	4	4	1	1	274	8695

### *Cryptosporidium* in marsupials

Within the *C. fayeri* clade, 13 variants were recorded among a total of 57 *SSU* sequences. Within the *C. macropodum* clade, five variants were detected among a total of 116 *SSU* sequences obtained from a total of 6014 samples from macropods (94.1% from eastern grey kangaroos; Fig. 2; Tables 2 and 3). Among the positive *Cryptosporidium* detections, nearly all were from kangaroos, with the exception of two wallabies that carried *C. macropodum* v2. The first record of *C. ubiquitum* in a macropod was from a kangaroo at Winneke, Victoria (accession no. PV658885), while a single variant of *C. ubiquitum* (represented by accession no. PV658884) was detected among 217 wombats. In addition, one *Cryptosporidium* sp. possum genotype was detected from seven brush-tailed possum samples. Notably, a *C. parvum*-like sequence (accession no. PV658876) was detected in a wallaby, with two nucleotide sites displaying multiple peaks that distinguish it from typical *C. parvum* sequences. Unfortunately, the *gp*60 region did not amplify from this sample. *Cryptosporidium* was not detected in the sole koala sample tested.

### *Cryptosporidium* in other animals (canids, lagomorphs, rodents and birds)

Of the 39 canid samples tested, *C. canis* was detected in a fox from Greenvale and a dingo from Cardinia (Table 3). From the 82 rodents examined, three tested positive for *C. viatorum* [21] and one for a variant of *C. occultus –* confirmed by the alignment criteria set forwards from Stensvold et al. [38] (Table 3). Of the 198 samples from rabbits tested, five were positive for *C. cuniculus* (Table 3). Of these, two samples were sub-typed using the *gp*60 gene sequence: the first sequence (accession no. PV665486) defined subtype VbA31R4 originating from OS (December 2018) but with one point mutation over a 300 bp region when compared with the GenBank sequence accession no. KU852732 (822 bp) representing *C. cuniculus* from a human from Greece; the second sequence (accession no. PV665487) defined subtype VbA15, from YY. Of the 223 samples from waterbirds (predominantly wood ducks) tested, four contained *C. baileyi* (Table 3), but none of the 106 samples from emu tested positive for *Cryptosporidium*.

### *Giardia*

*Giardia* was detected in 14 of the 8695 faecal DNA samples tested (Table 4). *G. duodenalis* was identified in seven deer – six belonging to sub-assemblage AIII and one to sub-assemblage AI. In eastern grey kangaroos, sub-assemblage AI of *G. duodenalis* was detected in five individuals, including four novel genetic variants (GenBank accession nos. PV665481–PV665484). *Giardia* sub-assemblage AI was also detected in one dog. No *G. duodenalis* was detected in rabbits, rodents or birds.

**Table 4:** Total count of *Giardia* by catchment and host

Catchment	Canid	Deer	Emu	Koala	Macropod	Possum	Rabbit	Rat	Unknown	Waterbird	Wombat	Total
Armstrong												
Cardinia		4 (1 AI, 3 AIII)										4
Greenvale					2 (2 AI)							2
Maroondah					1 (AI)							1
O’Shannassy	1 (AI)											1
Silvan					1 (AI)							1
Upper Yarra												
Winneke												
Yan Yean		3 (2 AI, 1 AIII)			1 (AI)				1 (AI)			5
Total	1	7			5				1			14

### Coinfections

Both *Cryptosporidium* and *Giardia* were detected in a small number of samples (*n* = 3). *Cryptosporidium* sp. deer genotype v3 and *G. duodenalis* subtype AIII were detected in a red deer at YY in the summer of 2018, and *Cryptosporidium* sp. deer genotype v1 and *G. duodenalis* subtype AI were detected in a fallow deer at CA in autumn 2021. In addition, *C. fayeri* v2 and *G. duodenalis* subtype AI were identified in one kangaroo in the SV catchment in the summer of 2020.

### Temporal effects

**Fig. 3:**
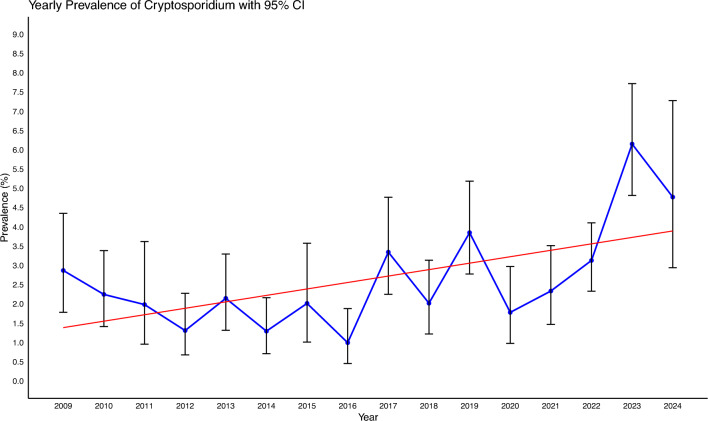
Prevalence of *Cryptosporidium* in animals in Melbourne’s protected drinking water catchments by year since 2009 – with 95% confidence interval bars

*Cryptosporidium* prevalence ranged from a low of 0.99% in 2016 to a high of 6.15% in 2023, with an overall mean prevalence of 3.15% (274 records among 8695 samples). When data from 2009 onwards was considered, the total prevalence across all 16 years was 2.67% (399 cases out of 14,960 samples) (Fig. 3). A statistically significant increase in prevalence was seen across years (adjusted R-squared 0.294; *P* = 0.0175).

**Fig. 4:**
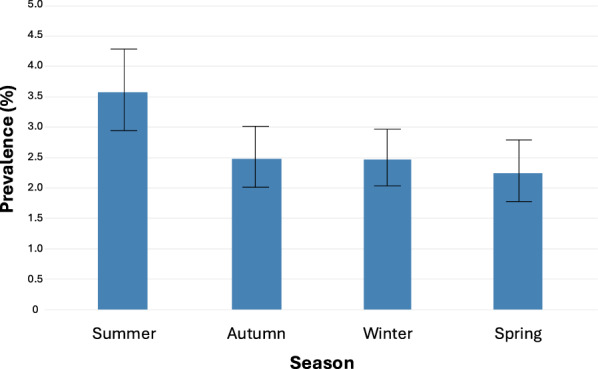
Prevalence of *Cryptosporidium* in animals in Melbourne’s protected drinking water catchments by season since 2009 – with 95% confidence interval bars

**Fig. 5:**
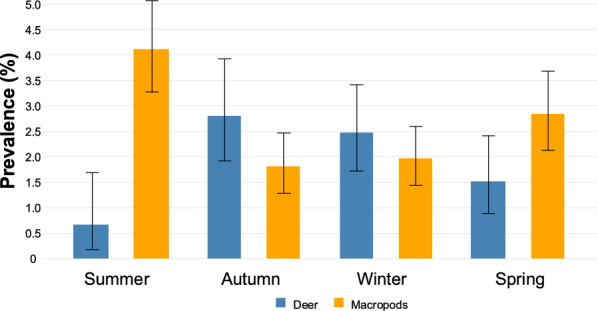
Prevalence of *Cryptosporidium* in macropods and deer – by season – in Melbourne’s protected drinking water catchments since 2009 – with 95% confidence interval bars

Over the entire period from 2009 to 2024, *Cryptosporidium* prevalence was the lowest in spring at 2.24% (78 records in 3482 samples) and the highest in summer at 3.57% (110 records from 3078 samples). Prevalences in autumn and winter were 2.48% (96 records from 3877 samples) and 2.47% (110 records from 4460 samples), respectively (Fig. 4). According to host groups and season, macropods had the highest *Cryptosporidium* prevalence in summer (4.10%; in 81 of 1976 samples) and spring (2.83%; in 53 of 1873 samples), with lower prevalences in autumn (1.80%; in 38 of 2106 samples) and winter (1.95%; in 47 of 2405 samples). In contrast, deer exhibited the highest *Cryptosporidium* prevalences in autumn (5.47%; in 32 of 1145 samples) and winter (2.47%; in 35 of 1418 samples), with lower prevalences in spring (1.51%; 17 of 1123 samples) and summer (0.67%; 4 of 600 samples) (Fig. 5).

**Fig. 6:**
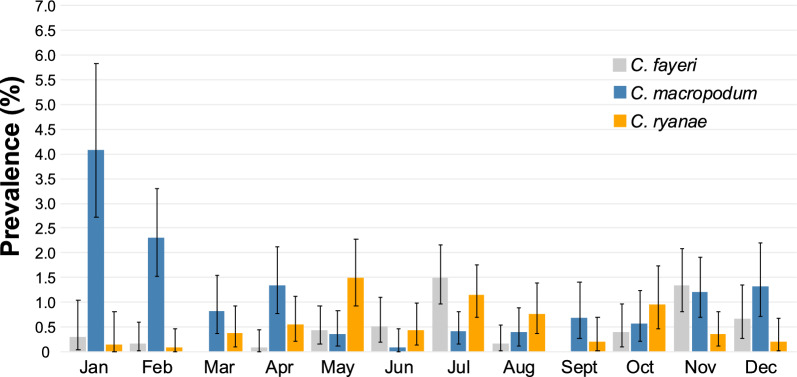
Prevalences of *Cryptosporidium fayeri*, *C. macropodum* and *C. ryanae* in animals – by month – within Melbourne’s protected drinking water catchment system since 2009 – with 95% confidence interval bars

An analysis of the monthly prevalence across all years for the three dominant *Cryptosporidium* species (*C. fayeri*, *C. macropodum* and *C. ryanae*) revealed distinct seasonal trends. *C. macropodum* exhibited a peak prevalence in the summer month of January, with the lowest prevalence recorded in June. In contrast, *C. fayeri* and *C. ryanae* showed peak prevalences in the winter months (Fig. 6).

## Discussion

Ensuring safe drinking water from natural catchments requires effective risk management and an understanding of protozoan parasite populations in the wildlife found in these areas. Melbourne Water’s chlorination treatment targets bacterial and viral pathogens but is not designed to remove the key protozoan parasites – *Cryptosporidium* and *Giardia* – which can pose health risks that are not always treatable with current chemotherapeutic options. Thus, ongoing surveillance of parasitic pathogens in catchments has been essential. This longitudinal study provides a strong and consistent foundation for understanding *Cryptosporidium* and *Giardia* populations in Melbourne’s catchment environments. Given that these parasites cannot be accurately identified or readily cultured with conventional laboratory techniques, this study employed cost-effective molecular genetic methods for their genetic identification and characterisation.

Here, we report the most recent results from one of the largest, continuous and most comprehensive longitudinal investigations of *Cryptosporidium* and *Giardia* in animals inhabiting protected drinking water catchment areas, serving the major metropolitan city of Melbourne in Australia. Nonetheless, smaller studies have been conducted in Victoria, New South Wales, South Australia, Queensland and Western Australia [39–46]. Overall, the prevalence of these two genera of protozoan parasites was relatively low (3.15% for *Cryptosporidium* and 0.16% for *Giardia*), with some fluctuations in prevalence from 2009 to 2024 and some spikes of *Cryptosporidium* in summer 2023. The prevalence of *Giardia* was extremely low, with sub-assemblages AI and AIII detected (assemblage A). Notably, four novel AI variants were identified. Sub-assemblage AI was present in deer, macropods and a canid, whereas AIII was exclusively found in deer – in accord with previous studies overseas [10, 47]. However, the low number of *Giardia* detections limits our ability to draw major conclusions from these data.

The low prevalences of *Cryptosporidium* and *Giardia* recorded compared with surveys in other states of Australia [39–46] might relate to our much larger sample sizes, the host species assessed, variation in habitats and/or catchment management practices, including the control or removal of animals from catchments. Although *Giardia duodenalis* assemblage AI (accession no. PV665479) has recognised zoonotic potential and was detected at a low prevalence (Table 4), *Cryptosporidium* remains the predominant concern for the water industry, as waterborne cryptosporidiosis in humans is typically far more challenging to manage and treat – particularly in immunosuppressed or immunodeficient individuals [3, 48].

Using PCR-based sequencing-phylogenetic analysis, we recorded 12 recognised species and genotypes of *Cryptosporidium* (Table 2). Nine species, known to occur in humans (*C. canis*, *C. cuniculus*, *C. fayeri*, *C. muris*, *C. occultus*, *C. parvum*, *C. ubiquitum* and *C. viatorum*) were recorded here in canids, deer, rats, rabbit, kangaroo, wallaby or wombat. Of the 46 recognised species of *Cryptosporidium* [8, 9], *C. hominis* and *C. parvum* are the most likely to cause human cryptosporidiosis [49] and are usually the key agents linked to waterborne outbreaks [15]. Despite their significance, the lack of *C. hominis* and the very low prevalence of *C. parvum* (0.01%) in the vast natural catchments (Table 3) suggests a negligible risk of waterborne transmission to humans in Greater Melbourne. Nonetheless, it is possible that some of the other *Cryptosporidium* species and genotypes discovered might have zoonotic potential.

### *Cryptosporidium* in deer

Two variants of both the ‘*Cryptosporidium* sp. deer genotype’ and a closely related *C. ryanae*-like genotype make up the majority of sequences found in the deer from this study (68/74 or 92%). An exact match to the distinctive *SSU* sequence (accession no. EU410344) representing *C. ryanae*, typically found in cattle, was not detected in this study, which is not unexpected, given that sampling sites were distant from cattle properties. The *Cryptosporidium* sp. deer genotype has been found in deer around the world including Père David’s deer, red deer, roe deer, sika deer and white-tailed deer [37, 50–52] – reviewed in refs. [53–55]. In China, the *Cryptosporidium* sp. deer genotype is quite common [53, 56] and has only been reported from deer [53]. Similar to the present study, both Dashti et al. [54] and Jäckal et al. [55] observed high genetic variability among *Cryptosporidium* sp. deer genotype samples in large studies of roe and red deer in Germany and Spain, respectively. Jäckal et al. [55] identified eight variants with at least one bp difference, while Dashti et al. [54] also reported variability, though their sequences were limited to ~450 bp in length. It is not understood why some *Cryptosporidium* species and genotypes have limited sequence variability while others tend to have greater variation within *SSU*. One possible explanation is that the introduction of multiple deer species into Australia from geographically isolated populations may have contributed to this diversity. Further exploration using *gp*60 and other markers may provide more insight.

Only one sambar deer from the Upper Yarra catchment tested positive for *C. ubiquitum.* This species had been detected previously on three occasions in a prior study at O’Shannassy and Cardinia, where three samples tested positive [18]. Commonly reported in ruminants and rodents [8], we identified identical sequences in both the deer from Upper Yarra and a kangaroo from Winneke (discussed hereafter). As its name suggests, *C. ubiquitum* has a broad host range and has been detected in deer populations worldwide [56, 57], indicating its adaptability and potential for cross-species transmission.

*Cryptosporidium muris* was detected in sambar deer at Cardinia and Yan Yean in 2021, and again at Yan Yean in 2023, representing the first recorded occurrences of this species in Melbourne’s catchments. While being named for and documented in a wide range of rodents [58], *C. muris* has also been reported in humans, pigs, pigeons, camels, black-boned goats, sheep, horses and various captive zoo animals [42, 59]. This species was also found in red deer and white-tailed deer at a game preserve in the Czech Republic [52]; mechanical transmission via contaminated feed was suspected as the source of infection.

Two samples tested positive for *C. occultus* from the same locality (Upper Yarra) in 2019 and 2024 (5 years apart), one from a deer and the other from a broad-toothed rat (*Mastacomys fuscus*) (accession nos. PV668769 and PV658875). These novel sequences were very similar, differing by only a single nucleotide, and have been designated as *C. occultus* rather than *C. suis* – in accordance with the guidelines provided by Stensvold et al. [38]. Reports of *C. occultus* in ruminants such as cattle, yak and water buffalo could result from the ingestion of rat faeces in contaminated feed or water, and subsequent gastrointestinal (‘mechanical’) passage [60], which could similarly account for its presence in deer.

### *Cryptosporidium* in marsupials

Within the *Cryptosporidium fayeri* clade, we identified 13 variants among 57 sequences, with three variants being the most prevalent: *C. fayeri*, *C. fayeri* EGK1 v4 and *C. fayeri* KG1-like (Fig. 2). Attempts to amplify the *gp*60 gene using primers from Power et al. [61] were unsuccessful, possibly owing to excessive variability at the primer-binding sites. The high genetic diversity observed within the *C. fayeri* clade may reflect a long-standing history of *Cryptosporidium* diversity among macropod marsupials, which – through host switching – has become consolidated within contemporary eastern grey kangaroo populations. Notably, this species of kangaroo, in this study alone, hosts at least 18 variants of *Cryptosporidium* representing three distinct species (*C. fayeri*, *C. macropodum* and *C. ubiquitum*), highlighting their unique role as hosts. Marsupials are already known for their exceptional diversity of helminth parasites [62], and this trend of high diversity appears to also extend to *Cryptosporidium* in kangaroos.

When *C. fayeri* was described by Ryan et al. [63], sequences EGK2, K1 and K2 were all designated as *C. fayeri*, whereas EGK1 was classified as a subtype of *C. fayeri*. Later, Yang et al. [64] first reported ‘Kangaroo genotype 1’ (11 sequences), none of which were deposited in GenBank. These sequences are more closely related to the ‘goose’ and ‘deer’ genotypes. Subsequently, additional samples from the latter study, which were genetically identical to *C. fayeri*, were deposited as ‘Kangaroo genotype 1’, leading to ongoing confusion. A sequence comparison with the original ‘Kangaroo genotype 1’ revealed no matches to any samples identified in this project. This genotype might be specific to macropods in Western Australia, and further investigation is warranted to address this proposal.

Most *SSU* sequences representing *C. macropodum* were dominated by a single sequence, all originating from kangaroos and had previously been classified under marsupial genotype II before its formal description [65]. In two wallabies, we identified a wallaby-specific subtype of *C. macropodum* (v2), previously detected in six wallabies from the O’Shannassy, Silvan and Cardinia reservoirs [18]. This particular sub-genotype was found exclusively in wallabies from Silvan and Yan Yean. None of the other 299 molecularly confirmed wallabies in this study tested positive for *C. macropodum*. Brush-tailed wallabies from New South Wales have also been reported to harbour this variant [66].

A wombat and a deer at Upper Yarra and a kangaroo at the Winneke treatment plant were found to host *C. ubiquitum*. This marks the first confirmed record of *C. ubiquitum* in a macropod, as previous reports of *C. ubiquitum* in rock wallabies were later identified as *C. macropodum* [18, 66, 67]. In addition, a *C. ubiquitum*-like sequence was identified in a wombat from Upper Yarra. This sequence has been observed on two prior occasions in wombats from Cardinia and O’Shannassy and may be specific to wombats [20].

Only seven possum faecal samples were collected, and one tested positive for *Cryptosporidium* sp. possum genotype. This genotype has been seen previously but the sequences on GenBank are mostly derived from cloned amplicons [68, 69], making the authenticity of the variants difficult to discern. Further exploration into this genotype would be beneficial, as brushtail possums are very common in Australia’s metropolitan areas [70].

The wallaby sample from O’Shannassy, collected in summer 2018, was classified as *C. parvum*-like on the basis of the presence of two multi-peak sites on the chromatogram (GenBank accession no. PV658876). A similar sequence, exhibiting the same features, was previously recorded in cattle from Turkey (GenBank accession no. MT416398, unpublished). Unfortunately, amplification of the *gp*60 region was not successful for this sample. Notably, O’Shannassy has experienced increased human activity owing to dam wall reconstruction from 2018 to 2022.

### *Cryptosporidium* in other animals (canids, lagomorphs, rodents and birds)

Of the 39 canid samples, two tested positive for *C. canis*. The more interesting one was from a family of dingoes that was spotted in the Cardinia reservoir with the aid of a trail camera. Over the years, 121 canid samples have been examined, mostly from dogs and foxes. In Australia, a previous study involving dingoes and wild dogs from Sydney’s drinking water catchment found a prevalence of 22.7% (*n* = 44) [41]. Future work determining the *gp*60 sub-typing is warranted [71] to see if species-specific genotypes of *C. canis* exist in foxes, dogs and dingoes.

For *C. cuniculus*, we were only able to sequence *gp*60 from two out of the six samples that were positive by *SSU*: VbA31R4 and VbA15. This is the first time that these subtypes were recorded in the catchments, and each has been recorded from both humans and rabbits previously [58]. The rabbit sample from OS in December 2018 had 1 bp difference over 300 bp compared with the sequence with accession no. KU852732 obtained from a human from Greece. Over the years, there has been a steady decline in *C. cuniculus*, which could be owing to sampling effort (finding fresh rabbit faeces) or owing to the rise and fall of rabbit populations in the catchments.

*Cryptosporidium viatorum* was originally detected in 2012 in the UK from travellers returning from India [72]. The first detection of *C. viatorum* (XVbA2G1) from rodents was in 2015 from Australian swamp rats (*Rattus lutreolus*) at Upper Yarra and then again in 2017 [21]. Since then, multiple studies have detected *C. viatorum* in a variety of rodents and insectivores from China [38, 58, 73, 74]. A different subtype of *C. viatorum* (XVaA3g) has also been detected in Australian human from Western Australia [75]. The floodplains at Upper Yarra, where the samples were collected, have undergone controlled flooding in recent years, leading to the gradual regeneration of habitat suitable for rats.

The majority of the waterbird faecal samples collected are most likely from the Australian wood duck (*Chenonetta jubata*), the most prevalent species in the catchments. In previous studies, we detected 3 out of 56 samples examined were found to have *Cryptosporidium* sp. duck-like genotype [18], while no *Cryptosporidium* was detected in 29 samples during the earlier study by Nolan et al. [17]. For the first time, we detected four samples positive for *C. baileyi,* despite it being commonly reported from wild birds [76]. The absence of *Cryptosporidium* in emus from the Cardinia catchment is not surprising, as it was rarely detected in previous studies [18]. In addition, the emu population here has been isolated since they were introduced in the late 1980’s [77], and they do not occur in any of the other catchments.

### Comparisons with Sydney’s and other water catchment systems

Over the years, several studies have investigated *Cryptosporidium* in wildlife from water catchments. Aside from Melbourne’s catchments, the most extensively studied has been Sydney’s water catchment [39, 41, 42, 45, 78, 79]. The most comparable host species between the two catchments are kangaroo, as deer were infrequently sampled from Sydney’s catchments. Unlike Melbourne’s closed catchment system, samples from Sydney were collected from both inner catchments, where native animals dominate, and outer catchments, which have high numbers of livestock animals and wildlife [41]. In studies by Power et al. [39, 79], a prevalence of 6.72% (239/3557) was reported. This prevalence was determined using immunomagnetic separation (IMS), flow cytometry (FC) and immunofluorescence assay (IFA), not PCR. Subsequently, 51 positive samples were sequenced and identified as either *C. fayeri* or *C. macropodum* (previously referred to as marsupial genotypes I and II). Cox et al. [78] reported a prevalence of 19% in a small survey of 10 kangaroos and 11 wallabies, but no PCR-based sequencing was performed. Ng et al. [41] examined 160 kangaroo faecal samples from 2009 to 2011 and found a prevalence of 16.9%: 18 *C. hominis,* 6 *C. parvum*, 2 *C. macropodum* and 1 *C. parvum*-like. From July 2013 to December 2015, Zahedi et al. [42, 45] examined 835 kangaroos and found a prevalence of 8.6% (72/835) consisting of 24 *C. parvum*, 17 *C. hominis*, 2 *C. macropodum* and 2 other. When compared with macropods examined over the 16 year period from Melbourne’s catchments, there was a prevalence of 2.65% (221/8342) (Additional File 1: Supplementary Table S2), the majority of which were *C. macropodum* and *C. fayeri*. The differences in prevalence may be attributed to detection methods; however, the composition of *Cryptosporidium* species varies markedly between the two catchments. The dominance of *C. parvum* and *C. hominis* reported by Ng et al. [41] and Zahedi et al. [42, 45] highlights the influence of human activity and agriculture in Sydney’s open catchments. In contrast, Melbourne’s closed catchment system showed very few detections of *C. hominis* and *C. parvum*, with *C. macropodum* and *C. fayeri* being far more prevalent (Table 3).

In Western Australia, a major study of *Cryptosporidium* in kangaroos revealed a prevalence of 15.2% (364/2393), of which 59.3% were *C. macropodum* and the remainder classified as ‘other’ [45]. In South Australia, Swaffer et al. [43] investigated *Cryptosporidium* in open water supply catchments by testing water samples using next generation sequencing. The majority of the samples were from *C. parvum*, *C. ubiquitum*, *C. tyzzeri*, *C. bovis* and *C. ryanae*, most likely from livestock and rodent sources [43].

### Temporal effects

*Cryptosporidium* prevalence was variable between 2009 and 2020 and then increased, peaking in 2023 (Fig. 3). One explanation for this increase from 2020 to 2023 could be the consecutive La Niña weather events that the eastern part of Australia experienced, bringing mild winters and overall increased precipitation [80]. It should be noted that the coronavirus disease 2019 (COVID-19) pandemic prevented us from collecting for several months in 2020 and 2021 owing to the mandatory lockdowns imposed on Melbourne. Efforts were made to make up for missed sampling in between lockdowns.

### Seasonal effects

When the seasonality of all *Cryptosporidium* species was examined, there was a distinct increase in prevalence during the summer months. No major difference can be seen when all *Cryptosporidium* species are examined across the 16 years. However, when they are broken down by the two most common host groups (macropods and deer), then seasonal effects can be observed. Deer have increased prevalence during the autumn and winter while macropods peak in the summer and spring. When *C. fayeri*, *C. macropodum* and *C. ryanae* are further separated out, our findings showed that *C. macropodum* peaks in the summer, *C. fayeri* is higher in winter and spring, and for deer, *C. ryanae* was higher in the autumn and winter months. Power et al. [39] examined *Cryptosporidium* in eastern grey kangaroos in Sydney’s water catchments and saw an increase in *C. macropodum* in winter while *C. fayeri* rose during the summer months which contrasted Thompson [81] who reported western grey and red kangaroos with *C. macropodum* in summer. When looking at seasonal patterns of *Cryptosporidium*, it is important to take into account the seasonal pattern of birthing/calving. The two dominant hosts in this study are deer and kangaroos. The sambar deer present in the catchment were introduced in 1862 from Sri Lanka [82]. According to Watter et al. [83], sambar deer in alpine and sub-alpine Victoria have a seasonal pattern of calving at 36° south, with approximately 80% of hinds predicted to calve during the 5 months between April and August; however, this seasonal pattern is not as distinct as that of animals of many other species. For eastern grey kangaroos, births are concentrated in the summer months in south-eastern Australia, with the young remaining in the pouch for 10 months [84, 85]. Age studies would need to be conducted to further investigate. Correlating *Cryptosporidium* prevalence in Australian wildlife is not as straight forward as it is with livestock.

## Conclusions

This article presents findings from the last 9 years of a longer 15-year longitudinal study investigating the occurrence and distribution of *Cryptosporidium* and *Giardia* in Melbourne’s water catchments. Analysis of 8695 faecal samples collected from a variety of wildlife hosts revealed that *Cryptosporidium* was present in 3.15% and *Giardia* in 0.16% of the samples. There were 12 recognised species/genotypes of *Cryptosporidium* (9 of these species, known to occur in humans), and 2 sub-assemblages of *Giardia* along with numerous previously unrecorded variants. Since the zoonotic potential of these novel genotypes remains unknown, future research should investigate their presence in humans across Victoria, Australia, and globally. Notably, the prevalence of the two main human-infectious species of *Cryptosporidium* was extremely low, indicating that they do not pose a major waterborne disease risk to the human population in Greater Melbourne. While the study focused on Melbourne’s drinking water catchments, its findings have broad implications for protected wilderness catchments worldwide that supply unfiltered drinking water. The improved knowledge from this long-term surveillance is crucial for designing integrated monitoring programs to safeguard water quality. Advancing parasite and host metagenomic sequencing, along with high-throughput analytical platforms, will further enhance catchment management – an essential priority amid climate change, with significant benefits for public health, industry and the economy.

## Supplementary Information

Supplementary material 1. Table S1. Summary of samples positive for *Cryptosporidium* and *Giardia* collected from Melbourne’s drinking water catchments from 2015 to 2024. Table S2. Numbers of faecal samples and *Cryptosporidium* test-positive samples from animals in Melbourne’s drinking water catchment system – by host – from 2009 to 2024

## Data Availability

Nucleotide sequences reported in this paper are available in the GenBank database under accession nos. PV658852–PV658885; PV665479–PV665488 and PV668769.

## Abbreviations

AR: Armstrong weir
CA: Cardinia
*gp*60: 60-KDa glycoprotein
GV: Greenvale
*LSU*: Large subunit ribosomal ribonucleic acid gene
MR: Maroondah
OS: O’Shannassy
*SSU*: Small subunit ribosomal ribonucleic acid gene
SV: Silvan
TH: Thomson
tpi: Triose-phosphate isomerase
UY: Upper Yarra
WI: Winneke
YY: Yan Yean
